# The effect of social networks on academic self-handicapping with the mediating role of self-regulatory learning strategies and academic achievement among EFL students

**DOI:** 10.3389/fpsyg.2022.987381

**Published:** 2022-12-13

**Authors:** Siros Izadpanah, Mahdis Charmi

**Affiliations:** Department of English Language Teaching, Zanjan Branch, Islamic Azad University, Zanjan, Iran

**Keywords:** academic achievement, academic self-handicapping, self-regulatory learning social networks, social networks (online), education

## Abstract

**Introduction:**

In the academic context, social networking sites (SNSs) have reshaped the way university students connect and communicate with each other and the way they learn, thus influencing their identities and dimensions. This research investigates the effect of social networks on academic self-handicapping with the mediating role of self-regulatory and academic achievement.

**Method:**

The current study is considered to be quantitative research in terms of its nature, applied research in terms of its purpose, and correlational descriptive one in terms of its method. The statistical population of the present study includes all language students of Tabriz University, whose number was 540 people. Sampling was done by the random sampling method. Using Morgan’s table, the sample size was estimated to be 225 people in 2022. Due to the possibility of falling, 10% was added to this number, and 250 questionnaires were distributed among 250 participants; 17 questionnaires were incomplete, and a total of 233 valid questionnaires were examined. Four questionnaires were administered to collect data. SPSS 26 and AMOS 24 software were used to analyze the collected data from questionnaires. Descriptive, inferential, and Structural Covariance Matrix (SEM) statistics were conducted to explore the effect of social networks on these variables.

**Results:**

Findings showed that social networks significantly impact self-handicapping in language students of Tabriz University. 2- Social networks have a significant impact on self- self-handicapping with a mediating role of self-regulation learning strategies in language students of Tabriz University. 3- Social networks significantly affect self-handicapping, mediating role in language students’ academic progress at Tabriz University.

**Discussion:**

Further investigation and experimentation into social networks are strongly recommended. In addition, studies show that self-handicapping is successful in helping individuals divert the assessments of others from attributing weak abilities to failure. Because self-handicapping behavior undermines achievement and can lead to long-term withdrawal from activities (such as school and college), parents and teachers must discourage self-handicapping and avoid behavior that may encourage it.

## Introduction

The use of new communication technologies, especially social networks in recent decades, has brought us into a new era and society; the society that Daniel Bell called the post-industrial society, Tada Omsu, the network society, and Castell, the information society (Cueva and Inga, 2022). With the increasing development of electronic communication technologies such as satellite and Internet networks and their impact on many social, cultural, political, and economic aspects, society has undergone fundamental changes. Some scholars, including Joseph Ney, have called virtual social networks a new dimension of power in the 21st century (Ghasemzadeh, 2011; Fuhse, 2021). The main issue here is that despite all the advantages and disadvantages of using social networks, it should be acknowledged that many spatial aspects of virtual social networks and bases that work in the form of social media are still unknown, so the review of all aspects of these networks, especially checking their capabilities in the field of education, should be prioritized.

The overgrowth and widespread of SNSs have created concerns among communication experts, university authorities, and researchers about the usefulness and potential effect that university students face while engaging and using online networking sites (Yapıcı and Hevedanlı, 2014). SNSs have changed university students’ learning process (Geçer and Topal, 2015). Nowadays, university students worldwide tend to have more than one account on SNSs, which hinders their academic performance and impacts their lives and sociability. This vital issue has been widely addressed in many studies, and it has become prominent due to the many newly developed social sites such as Facebook, TikTok, WhatsApp, etc. (Aljaraideh and Al Bataineh, 2019; Tavakli and Mansour Lecourge, 2019; Albashtawi and Al Bataineh, 2020).

Self-handicapping behavior or choosing a set of behaviors allows a person to attribute failure to external factors and success to internal ones (Callan et al., 2014). Academicc self-handicapping is a defensive strategy in which a person creates barriers before an academic performance to manipulate their documents and act in a way that indicates their inadequacy (Uysal and Knee, 2012; Schwinger et al., 2022). Vrij et al. (2021) concluded that self-handicapping significantly correlates with high school students’ academic self-efficacy. With decreasing academic self-efficacy, self-handicapping increases and leads to negative psychological consequences. Kumari (2015), in her research, showed that academic self-handicapping reduces adolescents’ success and academic performance and leads to academic failure and eventual school dropout.

Currently, the evidence shows that to improve the progress and success of students in learning, it is possible to achieve the desired academic achievement by using cognitive interventions. Therefore, some interventions are needed to help students reduce negative perfectionism, self-handicapping, and academic failures, and appropriate identification and interventions are essential in this regard. Indeed, early intervention in self-handicapping should be an important focus for school mental health professionals. One of the most critical psychological interventions is the use of cognitive-behavioral coaching techniques, during which, in a collaborative process, the underlying thoughts or cognitive and fundamental schemas in the behavior and feeling are the focus of the intervention. The use of short-term, effective interventions, affordable and tailored, is necessary to get rid of negative perfectionism and academic self-handicapping.

Self-regulation is an active and constructive process according to which the learner considers a series of goals for their learning and then tries to control and adjust these goals and guide their cognition, motivation, and behavior (Bakker and de Vries, 2021). Although there are different definitions of self-regulation in other theories, in general, self-regulation is defined as the ability to control and regulate individuals’ learning using cognitive and metacognitive strategies (Trotter et al., 2021). Teng and Zhang’s research results show that decreased intrinsic motivation to learn and lack of self-regulation lead to increased procrastination (Teng and Zhang, 2022). Regarding the components of motivational strategies, the results also showed that self-efficacy, intrinsic value, test anxiety, and cognitive strategies are negative predictors of academic self-handicapping (Inzlicht et al., 2021).

Hypotheses:

1. Do social networks significantly affect self-handicapping in language students of Tabriz University?
2. Do social networks significantly affect self-handicapping with the mediating role of self-regulated learning strategies in language students of Tabriz University?
3. Do social networks significantly affect self-handicapping with the mediating role of academic achievement in language students of Tabriz University?

This study investigates the effect of social networks on academic self-handicapping with the mediating role of self-regulatory learning strategies and academic achievement among EFL students of Tabriz University.

## Review of literature

### Social networks

The term web-based social network was first introduced in 1960 at the University of Illinois in the United States. In 1997, the first social networking site, Six Degrees, allowed users to create profiles to produce a list of friends. The spread of the concept of business on social media in (2002) led to the emergence of social networks such as Esther Friend, Orkut, LinkedIn, and the mushroom growth of these sites in the virtual space. In (2006) public access to Facebook was released because before that; the site was only used as a pilot at Harvard University. Twitter also entered the social media arena in the same year (Sponcil and Gitimu, 2013). The concept of social networks in Iran became widespread among Iranian users around (2004) with the presence of the foreign network “Orkut,” and in a short time, it grew so fast that after Brazil and the United States, Iran became the third country in Orkut (Qarabaghi et al., 2017).

Social networks allow users to create informational content for publication on websites, blogs, and social networks. The user of social networks can be the creator of information, interpreter, or reviewer of information in the social community (Mahony et al., 2022). Social networks can also play a beneficial role in business growth through product research and development, sales, marketing, and customer service (Al-vari and Magaki, 2017; Chang et al., 2017). Using social networks provides various benefits to the user, such as facilitating cooperation between friends, expanding human communication, and increasing the productivity of companies to develop relationships with customers (Bakhshi et al., 2013; Forutani and Bahrani, 2018).

Unlike other new information technology phenomena, virtual social networks do not have a comprehensive definition. However, most scholars and researchers refer to virtual social networks as virtual places to communicate and share content that has affected all aspects of people’s lives. Virtual social networks strengthen and expand social connections in the Internet space. Users put information such as photos, personal information, interests, and workplace on their personal page, strengthening and expanding cyberspace communication (Alimoradi, 2010; Bour et al., 2020).

Virtual social networks are the types of social media that most closely resemble human society, and regardless of time, place, political and cultural constraints, they allow communication with many people (Yang et al., 2021). Therefore, the harms of using social networks, which have not been around for a long time, have become a topic of interest for many researchers. With the growth of virtual social networks, there is a serious concern about the excessive use and dependence of people, especially adolescents and young people, on these networks (del Coz et al., 2021). Research shows that approximately 301 million people worldwide suffer from extreme dependence on social networks; this dependence significantly disrupts a person’s daily activities (Ozimek and Förster, 2021). According to new theories, reliance on social networks refers to the excessive use of these networks, which causes restlessness in the absence of access and fear of the lack of these networks (Schuster et al., 2021). Internet addiction is associated with symptoms such as anxiety and obsessive thoughts, dependents have problems in their daily activities and cannot control their lack of access to the use of media and social networks (Kuss and Griffiths, 2015; Shestak et al., 2021).

Although the word network and community both refer to the pervasiveness and high penetration of these technologies in the world; the use of this technology in the student class (university)and then students(high school) is more than in other classes of society (Sponcil and Gitimu, 2013). According to research, almost all students have a user name in at least one social network, and the age range of users is between 13 and 23 years (Yang and Tung, 2007). Therefore, addiction to social networks is one of the problems that has plagued different communities (Chavoshzadeh, 2009). Numerous and various types of research have been done in this field, and some of its harmful effects have been revealed; the results of Chavoshzadeh’s study showed that too much time using the Internet and social networks causes academic problems and low grades for students.

### Self-handicapping

Self-handicapping is one of the basic constructs of educational psychology and a term that has been considered in psychology sources since 1960 (Kolditz and Arkin, 1982; Schwinger et al., 2022). Self-handicapping is a defensive strategy in which the person creates barriers before the performance to manipulate their masters after the performance. Self-handicapping can be both behavioral and assertive (claimed; Funkhouser and Hallam, 2022; Melhem, 2022). Behavioral self-handicapping is defined as intentional, observable, and often external actions that directly affect performance. Though assertive (claimed) self-handicapping is internal and accidental and does not necessarily reduce the chances of optimal performance, it is more limited to excuses (Fadhli et al., 2021).

Jones and Berglas (1978) are pioneers in this field. They have defined it as behavior or choice of a set of actions that provides an excellent opportunity for people to internalize failure and internal success. This structure has many consequences and has adverse effects on the educational system of any country (Jones and Berglas, 1978). Hence, many studies have identified the factors and processes that lead to self-handicapping and its consequences (Núñez et al., 2021). Since self-handicapping is an avoidant behavior that leads to reduced performance and academic degradation, affects a person’s personality and future adjustment, and hinders a person’s growth in adulthood, this category needs further investigation and attention.

In recent years, academic self-handicapping has been proposed as a defect in self-regulation in the inability to control one’s thoughts, emotions, feelings, and optimal performance by one’s criteria (Khaneghahi et al., 2022; Török et al., 2022). Therefore, self-regulation is one of the essential variables in explaining the cause of self-handicapping. So that many studies consider self-handicapping as a defect in learner self-regulation (Shin and Park, 2021). Theobald believes that learners who use self-regulatory learning strategies are less likely to commit self-handicapping (Theobald, 2021).

### Self-regulation

Self-regulation is an active and constructive process according to which the learner considers a series of goals for his learning and then tries to control and regulate these goals and guide his cognition, motivation, and behavior (Bakker and de Vries, 2021; Cengiz-Istanbullu and Sakiz, 2022). Although there are different definitions of self-regulation in other theories, self-regulation is generally defined as competence in controlling and regulating individuals’ learning using cognitive and metacognitive strategies (Khan et al., 2021). According to Pintrich and DeGroot, self-regulation includes two main categories of motivational beliefs and self-regulatory learning strategies (Braund and Timmons, 2021). Rox and Don’s research results show that decreased intrinsic motivation for learning and lack of self-regulation leads to increased self-handicapping (Rakes and Dunn, 2010).

Noting that motivation for academic achievement is particularly important, researchers have studied factors affecting it, including self-regulation. Self-regulation is an active and organized process in which learners choose goals for their learning, then try to regulate, control, and monitor their cognition, motivation, and behavior (Azevedo et al., 2022). One of the concepts raised in contemporary education is self-regulated learning.

It has practical consequences for learning, education, and success. Adaptation and success in school require that students expand and strengthen their cognition, emotions, or behaviors by developing self-regulation or similar processes to achieve their goals. Self-regulated learning is an active and self-sustaining process in which students control and regulate their cognition, motivation, results, behavior, and environment to advance their goals. Self-regulation is closely related to motivation. Self-regulation is a process through which students activate and maintain cognition, behaviors, and emotions that are systematically aimed at acquiring goals. Students who are motivated to achieve a goal engage in activities that they believe are useful. Self-regulation enhances learning, and the perception of greater competence maintains motivation and self-regulation to achieve new goals. In the social-cognitive theory, the type of learning that is called self-regulated learning considers learning from both the educational and educational aspects. In this method, learners will have personal control over the education process and therefore learn more quickly and accurately. Educational issues such as higher self-reliance, self-efficacy, and more responsibility will also be created in the learners. In learning, the learner’s self-regulation makes learning more efficient. It makes the person more self-reliant due to his careful supervision during the learning process and creates the necessary motivation for learning. The process of self-regulation in learning seeks to activate the learner and accept responsibility for his daily educational issues. By clarifying the personality characteristics of self-regulating people and other research that have been carried out regarding the formation of these characteristics, it is possible to help the motivation of students’ academic progress effectively and pay attention to its educational effects.

### Academic achievement

Students’ academic achievement is one of the critical indicators and owners of the efficiency of the educational system. Therefore the analysis of related factors is one of the most basic research topics in the education system (Chizoba, 2017 research; Wu et al., 2021).

The study and review of the research show that academic achievement is not the result of the influence of one factor, but several factors influence this variable. One of these factors is the motivation for academic achievement. The reason for academic achievement is one of the primary constructs presented to explain motivation in education (Emami Rizi, 2015; Madigan and Kim, 2021). Ins’ Slavin view (1983), the motivation for academic progress is the most critical motivation that educational psychologists deal with. The research of Madigan and Curran show that people are very different in terms of this motivation. Some are highly motivated and strive hard to achieve success in competition with others. Their work and others do not have much motivation to progress and succeed and are not ready to take risks to achieve success for fear of failure (Malmir et al., 2015 research; Madigan and Curran, 2021; Schwinger et al., 2022).

Therefore, according to the contents presented above, based on theoretical foundations and empirical background, it can be said that there is a relationship between the research variables in the current research.

One of the influential factors in the tendency of people to study and learn is the motivation for progress. This theory emphasizes the goal’s role in students’ success and failure. This factor plays a fundamental and vital role in learning and is the engine of a person’s movement for behaviors that lead to better and more effective learning (Wang et al., 2021; Papageorgiou, 2022). Motivation for progress means the desire or desire to achieve success and participate in activities in which success depends on personal effort and ability. Progress motivation is a driver that accelerates the learning of homework so that a person can become competent and achieve success.

In other words, the main issue raised in this research is whether self-regulation learning strategies and academic achievement can be effective as a mediating variable on the effect of social networks on academic self-handicapping.

## Theoretical backgrounds

### Social networks

(A) Internet
  Today, in the competitive and rapidly changing business environment, access to correct, timely, and relevant information plays a vital role; so many activities of organizations such as decision-making, forecasting, and business analysis depend on this information. Information technology is a tool that can meet the information needs of organizations and help them achieve their goals (Cueva and Inga, 2022).
  The Internet is a global system of interconnected computer networks that use standard Internet protocols to serve billions of users worldwide. The Internet is a network of millions of local or national secret, public, academic, commercial, and government networks connected by electronic, wireless, and optical technologies.
(B) The concept of virtual space
  Virtual space presents a unique environment of its own: an environment in which individuals and organizations are continuously producing, packaging, re-packaging, recording, discarding, modifying, transferring, disseminating, accessing, and using information (Wheatstone, 1838). Virtual space can describe all types of information resources created through computer networks. In fact, virtual space is a different type of virtual and digital reality provided by interconnected computer networks, which with a little tolerance can be considered synonymous with the global Internet network.
(C) The concept of social networks
  In the new world system, we live in the age of media, an era where mass media are an integral part of life. Meanwhile, new media and virtual social networks were able to influence and transform people with the change in culture and the emergence of modern culture.

One of these communities is virtual social networks. Virtual social networks are also a new concept that is used today as simply as the concept of natural communities. The idea of virtual communities was first proposed in 2014 by Ringold as a social group in the Internet environment through which people discuss with each other. Virtual communities are spaces where members about a topic, come together through sending messages, in which attempts are made to model physical places as well as face-to-face communication, and in which people, not information, have access to other people for discussion and exchange of ideas.

## Types of social networks

### Twitter

Twitter is a social network and microblogging service that allows users to send 140-character text messages called tweets.

Twitter was created in March 2006 by Dorsey and launched in July 2006. Microblogging had over 100 million users in 2012, sending 340 million daily tweets and over 1.6 billion searches.

### Facebook

Zuckerberg and some of his friends designed the Facebook website from his room at Harvard University and launched it in 2004 as a social website for Harvard students. This website was very popular among Harvard students as a network through which students could communicate with each other. For a while, this site was only used by students of Harvard University, but in March of the same year, this social network reached Columbia, Stanford, and other universities.

### YouTube

YouTube is the most popular social network for watching and sharing videos. This network was launched in 2005 by three PayPal executives, and the following year it was bought by Google for $1.6 billion.

### Instagram

Instagram is a visual social network owned by Facebook. This site was launched in Auto Cad 2010 and has more than 500 million active users. Most users use this network to send information such as travel, fashion, art consumption and similar topics.

### WhatsApp

WhatsApp is messaging software for smartphones, computers, and tablets. This application sends images, sound, video, and documents to other users who show the software on the system.

## Materials and methods

### Statistical population

The statistical population of the present study includes all language students of Tabriz University in the undergraduate course, whose number is 540 people. Sampling is done by the random sampling method. Using the Morgan table, the sample size was estimated to be 225 people. Due to the possibility of falling, 10% was added to this number, and 250 questionnaires were distributed; 17 were incomplete, and 233 valid questionnaires were examined.

### Design of study

The present study is quantitative research in terms of nature, applied research in terms of purpose, and correlational descriptive one in terms of its method.

### Instruments

#### Social networks

Social Networking Questionnaire: To collect data related to the use of social networks, Sadeghi (2017) social network questionnaire was used using a 5-point Likert scale. This questionnaire is one-component and has 20 items. The validity of this questionnaire has been confirmed in Sadeghi Amin’s research, and its reliability has been calculated to be 0.89.

#### Self-handicapping scale

Jones and Rodwalt’s (1982) self-handicapping test was used in this study. The scale is scored on a 6-point Likert scale (from strongly disagree = 1 to strongly agree = 6). The scale also includes three factors: negative mood, self-regulatory learning strategies, lack of effort, and apology. In Iran, it was reduced from 25 to 23 items, and factor analysis confirmed its validity. The scale’s validity was 86% with the retest method, and the internal consistency was 77%. The combination of negative mood with the inverse score of effort (lack of effort) indicates behavioral self-handicapping, and the combination of negative attitude with apology indicates alleged self-handicapping. The correlation of this scale with related structures such as apology and low effort was reported in a sample of 245 people to be 27%. Also, its internal consistency has been reported in different studies from 38 to 70%.

#### Self-regulatory learning strategies

Self-regulatory learning strategies questionnaire: Pantridge and DeGroot’s (1990) self-regulatory learning strategies questionnaire was used to collect data on self-regulatory learning. The questionnaire has a range of 5 and is coded from 1 to 5. The questionnaire includes three components of cognitive strategies, metacognitive control, and resource management with 22 items. The factor of cognitive strategies is measured by the sum of the scores of items 1 to 13, the factor of metacognition is measured by the sum of the scores of items 14 to 18, and the factor of resource management is measured by the sum of the scores of items 19 to 22.

Pantridge and DeGroot’s (1990) research confirmed the validity of this questionnaire, and its reliability was calculated by Cronbach’s alpha method of 0.81.

#### Academic achievement

In this study, the purpose of academic achievement is 48 questions of Pham and Taylor’s (1994) standard questionnaire. The range of 5 questionnaires is from very low with code 1 to very high with code 5. Self-efficacy (items 29, 30, 31, 32, 33, 34, 35, 36), emotional effects (items 12, 13, 14, 15, 16, 17, 18, 19), planning (items 1, 2, 3, 4, 8, 9, 10, 11, 40, 43, 44, 45, 46, 48), lack of outcome control (items 5, 6, 37, 38) and motivation (items 20, 21, 22, 23, 24, 25, 26, 27, 28, 39, 41, 42, 47). In Dartaj’s (2004) research, the content validity of this questionnaire was confirmed by professors. The validity of this scale was also confirmed by factor analysis. The reliability of the questionnaire was evaluated by Cronbach’s alpha method, the results of which were self-efficacy equal to 0.92, emotional effects equal to 0.73, planning equally to 0.93, lack of outcome control equal to 0.64, motivation equal to 0.73, and in total the questionnaire was equal to 0.74.

#### Reliability and validity of the questionnaire

Factor analysis: To use structural equations, factor analysis is required, the results of which are presented in the following table:

According to Table 1, the KMO values for social networks, self-handicapping, self-regulatory learning strategies, and academic achievement questionnaires are 0.772, 0.877, 0.940, and 0.808, respectively, which indicates that the data volume is suitable for factor analysis. Also, according to the amount of surface covered by Chi-square statistics (significance level), the Bartlett index for all variables and their dimensions is equal to (0.001), which is less than the 0.01 level and shows that the data have a reasonable correlation.

**Table 1:** KMO and bartlett test to evaluate the adequacy of sampling and data correlation.

**Variables**	**KMO**	**Bartlett’s Test of Sphericity**	***p*-value**
Social networks	0.772	852.151	*0.001*
Self-Handicapping	0.877	3870.468	*0.001*
Self-regulatory learning strategies	0.940	3441.595	*0.001*
Academic achievement	0.808	43031.235	*0.001*

### Reliability

To evaluate the reliability of the research questionnaires, Cronbach’s alpha coefficient was used, and the results showed that Cronbach’s alpha for social networks is 0.834, self-handicapping is 0.972, self-regulatory learning strategies is 0.700, and academic achievement is 873.

To analyze the data in two sections of descriptive statistics and inferential statistics, SPSS 25 and Amos 24 software were used.

The results of descriptive statistics in Table 2 show that the average social network variables is 56.378, self-handicapping is 64.957, self-regulatory learning strategies is 10.172, and academic achievement is 23.394. Also, the values of skewness and elongation are in the range (2-and 2), which indicates that the data distribution is almost normal.

**Table 2:** Central indicators and dispersion of research variables.

	N	Mean	Std. deviation	Minimum	Maximum	Skewness	Kurtosis
Social networks	233	56.378	10.331	29	92	−0.281	0.443
Self-handicapping	233	64.957	23.604	30	121	0.263	−1.076
Self-regulatory Learning strategies	233	64.606	10.172	45	85	0.268	−1.115
Academic achievement	233	150.189	23.394	100	185	−0.196	−1.207

### Defaults of using structural equation methods

#### Default 1: Kolmogorov–Smirnov test

To check the normality of the research variables, the Kolmogorov–Smirnov test is used; the results are as follows:

Table 3 shows that the *p*-value is greater than 0.05, and the assumption of normal data is accepted.

**Table 3:** Evaluation of normality of variables by Kolmogorov–Smirnov test.

	**Test statistic (K-S)**	***P*-Value**	**Result**
Social networks	0.149	0.200*	The distribution of the variable is normal
Self-handicapping	0.123	0.200*	The distribution of the variable is normal
Self-regulatory learning strategies	0.131	0.200*	The distribution of the variable is normal
Academic achievement	0.173	*0.119*	The distribution of the variable is normal

*Significance at the level of 0.05 (*p* < 0.05).

#### Default 2: Pearson correlation test

To investigate the correlation of research variables, the Pearson test was used as follows:

The results of the Pearson test in Table 4 showed that the correlation coefficient of the research variables shows that all coefficients are higher than 0.4 and significant at 0.05.

**Table 4:** Correlation between research variables.

	Social networks	Self-handicapping	Self-regulatory learning strategies	Academic achievement
Social networks	1			
Self-handicapping	0.742**	1		
Self-regulatory learning strategies	−0.685**	−0.663**	1	
Academic achievement	−0.676**	−0.657**	0.640**	1

**Correlation is *p*-value significant at the 0.01 level (2-tailed).

The path analysis method with Amos 24 software investigates the relationship between research variables. The research model is as follows:

The model was fitted in Amos 24 software. The results were obtained as follows.

Paying attention to the software output, the calculated value of 2χ is equal to 2.532, which is less than 3 concerning its degree of freedom, i.e., 1. The low value of this index indicates a slight difference between the conceptual model and the observed research data. The RMSEA value is equal to 0.072. GFI, NFI, IFI, RFI, and CFI indices are equal to 0.949, 0.951, 0.953, 0.900, and 0.952, respectively, which indicate a high fit.

In the following, the research hypotheses are examined.

*Hypothesis 1*: Do social networks significantly affect self-handicapping in language students of Tabriz University?

The results of this hypothesis are presented in Table 5.

**Table 5:** Investigating the relationship between social networks and self-handicapping with the mediating role of self-regulatory learning strategies.

	Path coefficients	*T*-Value	*P*-Value	Condition
Social networks→ Self-regulatory learning strategies→ Self-handicapping	−0.685*−0.222 = 0.152	*2.049*	<0.05*	Accept

*Significant at the level of 0.01 (*p* < 0.01).

**Figure 1:**
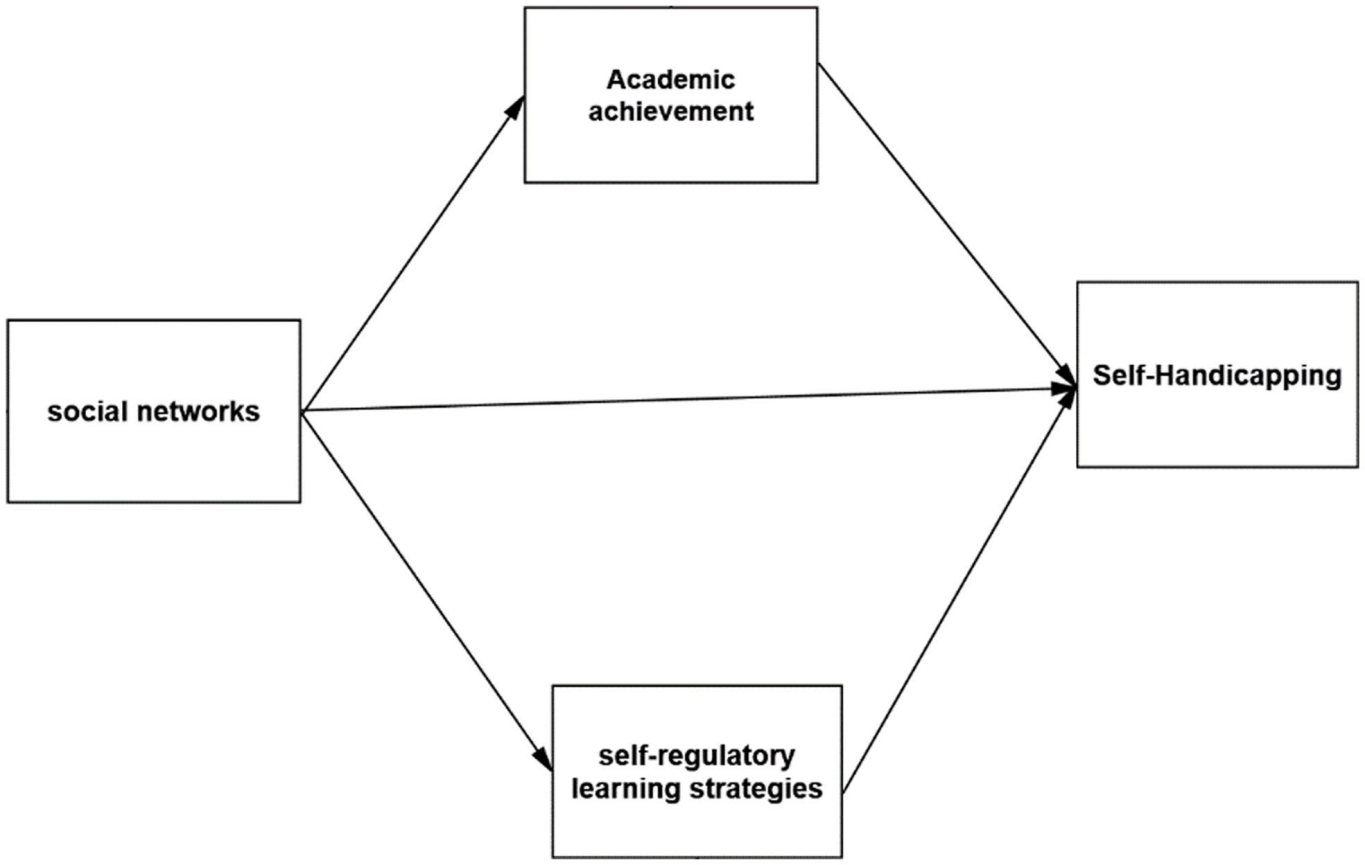
Research model.

The results of path analysis in Figure 1 and Table 5 show: The standard coefficient between social networks and self-handicapping is 0.450, and according to the absolute value of t-test statistics which is equal to 7.258 and greater than 1.96, it can be concluded with 99% probability that social networks have a positive and significant effect on self-handicapping (value of *p* = 0.001; β = 0.450). In other words, for one unit of social networks, self-handicapping increases by 0.450 units, and the coefficient of determination, which is equal to the power of the path coefficient, is 0.202, which shows that 20.2% of the self-handicapping changes are due to social networks.

*Hypothesis 2*: Do social networks significantly affect self-handicapping with the mediating role of self-regulatory learning strategies in language students of Tabriz University?

The results of this hypothesis are presented in Table 6.

**Table 6:** The relationship between social networks and self-handicapping with the mediating role of self-regulatory learning strategies.

	Path Coefficients	*T*-Value	*P*-Value	Condition
Social networks→ Self-regulatory learning Strategies→ Self-handicapping	−0.685*−0.222 = 0.152	*2.049*	<0.05*	Accept

*Significant at the level of 0.05 (*p* < 0.05).

**Figure 2:**
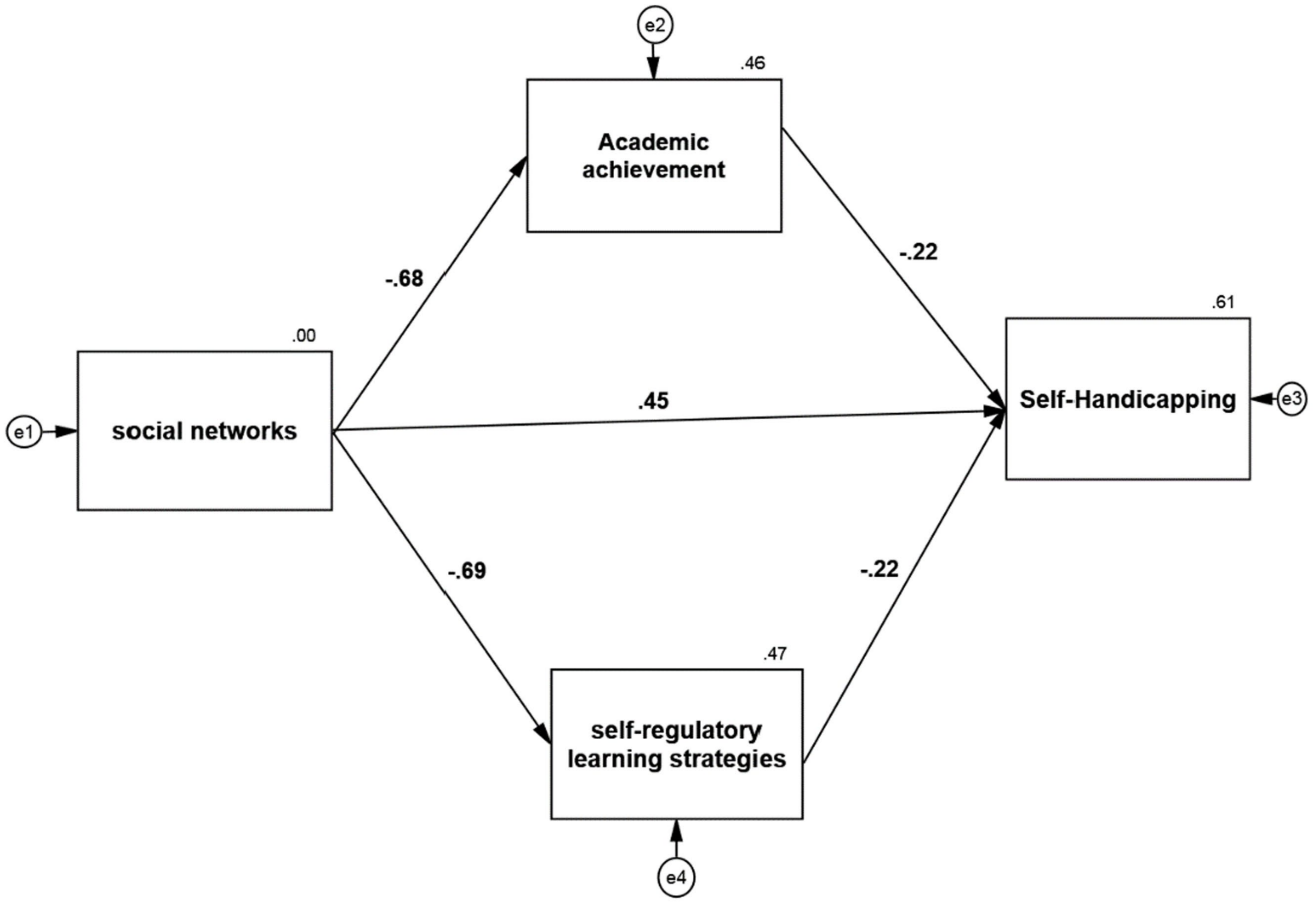
Model fit in standard estimation mode.

The results of examining the mediating role of self-regulatory learning strategies using the Sobel test in Figure 2 and Table 6 show that the standard coefficient between social networks and self-handicapping with the mediating role of self-regulatory learning strategies is 0.152. The Sobel test, equal to 2.049 and greater than 1.96, can be concluded with a 95% probability that social networks have a positive and significant effect on self-handicapping with the mediating role of self-regulatory learning strategies (value of *p* <0.05; 0.152 = b). In other words, in exchange for increasing one unit of social networks, self-handicapping with the mediating role of self-regulatory learning strategies increases by 0.152 units, and the coefficient of determination is equal to the power of the path coefficient, which is 0.023, which shows 2.3% of the changes. Social networks cause self-handicapping with the mediating role of self-regulatory learning strategies.

*Hypothesis 3*: Social networks significantly affect self-handicapping with the mediating role of academic achievement in language students of Tabriz University.

The results of this hypothesis are presented in Table 7.

**Table 7:** Investigates the relationship between social networks and self-handicapping with the mediating role of academic achievement.

	Path coefficients	*t*-value	*P*-value	Condition
Social networks→ Academic Achievement→ Self-Handicapping	−0.676*−0.216 = 0.146	3.114	<0.05*	Accept

*Significant at the level of 0.05 (*p* < 0.05).

**Figure 3:**
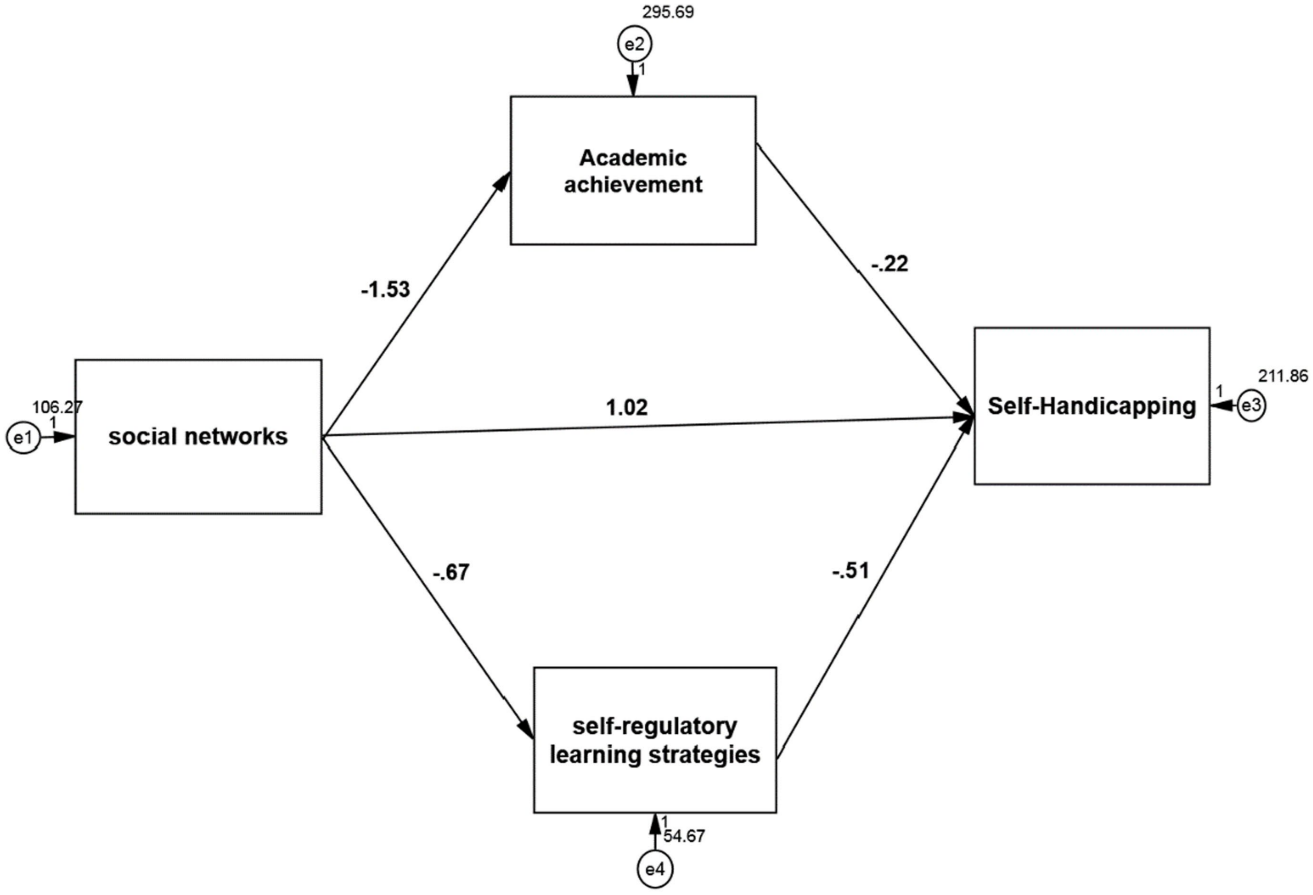
Model fit in non-standard estimation mode.

The results of examining the mediating role of academic achievement using the Sobel test in Figure 3 and Table 7 show that the standard coefficient between social networks and self-handicapping with the mediating role of academic achievement is 0.146. According to t-test statistics, the Sobel test, which is 3.114 and is greater than 1.96, it can be concluded that with a 95% probability, social networks have a positive and significant effect on self-handicapping with the mediating role of academic achievement (value of *p* <0.05; β = 0.146). In other words, in exchange for increasing one unit of social networks, self-handicapping increases by 0.146 units with the mediating role of academic achievement. The coefficient of determination, which is equal to the power of the path coefficient, is 0.021, showing that 2.1% of the self-handicapping changes are caused by social networks mediating academic achievement.

## Discussion

This study aims to investigate the effect of social networks on academic self-handicapping with the mediating role of self-regulatory learning strategies and academic achievement among EFL students of Tabriz University. Our research hypotheses are as follows:

(1) Do social networks significantly affect self-handicapping in language students of Tabriz University?

Findings showed a significant relationship between the use of social networks and students’ self-handicapping. That is, with students’ social networks, their academic self-handicapping increases. These findings are consistent with the empirical findings of researchers such as Uysal and Knee (2012), Callan et al. (2014), Fadhli et al. (2021), Vrij et al. (2021), Funkhouser and Hallam (2022), Schwinger et al. (2022).

When individuals fear or expect to fail in tasks that are important to them, they often engage in practices and behaviors that can increase the likelihood of failure (or at least lower achievement) so that they have an excuse for failure. This self-shooting can occur in any activity or domain. Academic self-handicapping has attracted a great deal of attention because academic achievement is reflected in an evaluative characteristic (represented as intelligence) and there are frequent opportunities for students to express their invisible abilities or shortcomings visibly. In other words, students often worry that they will look unintelligent if they are poorly tested on a test or task. Hence, they sometimes engage in self-handicapped behavior that provides an excuse for poor results.

Self-handicapped behavior is associated with lower achievement. Since self-handicapping behavior represents a reduction or withdrawal of effort toward a given task (non-preparation for a test), it is not surprising that the tendency to self-handicap is associated with less impact on those tasks.

These studies’ findings show that learners’ academic self-handicapping can be predicted based on the use of virtual networks. In explaining this finding, it can be said that one of the things that are effective in the process of doing tasks related to university and class is seriousness and tasks; despite the use of social networks by students, tasks are not done. This action leads to academic self-handicapping, which experts consider to be a very important problem in the academic achievement of students and learners. Chavoshzadeh’s research showed that too much time using the Internet and social networks causes academic problems and lowers students’ grades.

(2) Do social networks significantly affect self-handicapping with the mediating role of self-regulatory learning strategies in language students of Tabriz University?

In recent years, academic self-handicapping has been proposed as a defect in self-regulation in the inability to control the subject’s thoughts, emotions, feelings, and performance with the desired criteria (Schwinger et al., 2022). Therefore, self-regulation is one of the essential variables in explaining the cause of self-handicapping, so many researchers consider self-handicapping a defect in learner self-regulation (Funkhouser and Hallam, 2022).

Shin and Park (2021) believe that learners who use self-regulatory learning strategies are less likely to commit self-handicapping. Findings showed that the use of social networks has an indirect effect on students’ self-handicapping through the mediation of self-regulatory learning strategies. In other words, the results showed that self-regulatory learning strategies indirectly affect the relationship between social networks and students’ self-handicapping. Using social networks leads to increased academic self-handicapping. But due to the student’s use of self-regulatory learning strategies in the learning process, the effect is reduced. Therefore, self-regulatory learning strategies can play a reducing and moderating role in the relationship between the two variables as mediating variables. It shows a close relationship between social networks and academic self-handicapping, and the use of learning strategies can affect the relationship between these factors. The use of social networks in the learning process by learners makes the individual more efficient in learning. It plays an influential role in reducing the factors that reduce the quality of learning. By including the variable of self-regulatory learning strategies, the relationship between the use of social networks and academic self-handicapping change; because self-regulatory learning strategies increase the efficiency of the individual, which is in contrast to academic self-handicapping.

Self-regulatory learning strategies are valuable mechanisms for advancing learning goals (Fadhli et al., 2021; Teng and Zhang, 2022). Research showed a positive relationship between the use of virtual social networks and self-regulatory learning strategies and the components of self-regulatory learning strategies (cognitive and metacognitive strategies).

Theobald (2021) and Schwinger et al. (2022) also conclude that social media can facilitate the creation of personal learning environments and enable teachers to implement and develop self-regulatory learning processes such as setting appropriate personal goals, using work strategies to manage information, and engaging in self-monitoring. Self-assessment contributes to the advancement of social knowledge.

This study showed a significant relationship between self-regulatory learning strategies and students’ self-handicapping. If the student uses more self-regulatory learning strategies in the learning process, it will reduce academic self-handicapping and vice versa. This finding is in line with the experimental findings of researchers such as Bakker and de Vries (2021), Shin and Park (2021), Theobald (2021), Azevedo et al. (2022), Khaneghahi et al. (2022), Melhem (2022), Papageorgiou (2022), and Schwinger et al. (2022). Explaining this finding, we can say that self-regulatory learning strategies are opposed to academic self-handicapping; because self-regulatory learning strategies are considered to set a goal and plan to achieve the goal; self-handicapping, on the other hand, is delaying tasks or delaying important tasks by substituting trivial things. Therefore, the nature of these variables conflicts with each other. So, using self-regulatory learning strategies by the learner in the learning process reduces academic self-handicapping.

Matzat also showed in a study that the use of social media is naturally related to self-handicapping learning. It also showed that teachers use social media to share information with students, both in and outside the classroom, and that using social media to facilitate self-regulated learning does not affect teacher-student relationships (Chizoba, 2017).

Beyond what has been said above, which is related to the positive aspects of using social networks, other research results show that virtual networks do not lead to self-regulatory learning. In this regard, we can point to the factors affecting the relationship between these variables. One of the influential factors is Internet addiction and improper Internet use. Internet addiction is generally defined as Internet use that can cause psychological, social, academic, and occupational problems in a person’s life. Bakker and de Vries’s studies showed a negative relationship between Internet addiction and academic achievement. In other words, if people use the Internet or virtual (social) networks too much, it will decrease academic achievement. Therefore, it can be said that inappropriate use of the Internet and related issues such as virtual networks affect a person’s personality, which leads to disruption in life, the obvious signs of which can be a lack of attention to purpose, the value of time, and schedule—planning and managing available resources such as time, etc. Self-handicapping learning strategies are more related to issues such as goal selection, planning, and resource management to achieve the desired results (Schwinger et al., 2022).

3) Do social networks significantly affect self-handicapping with the mediating role of academic achievement in language students of Tabriz University?

The findings of this research show that the use of virtual social networks has positive effects on the academic achievement of language students. Results of Malmir et al. (2015), Emami Rizi (2015), Alwar and Maqam’s research (2017), Chizoba (2017), and Tazer et al. (2017) confirm this research result. The positive influence of social networks on improving academic achievement can be seen in the main features of social networks such as interactiveness, participation, and the possibility of using multimedia content. In virtual educational environments, people are responsible for their learning and are active, and these factors improve progress.

These findings align with the empirical findings of researchers such as Cengiz-Istanbullu and Sakiz (2022) and Papageorgiou (2022). Explaining this finding, it can be said that using social networks can produce different results depending on the purpose and type of application. In other words, just as excessive and inappropriate use leads to unwanted and harmful effects on people’s performance, in contrast, its purposeful and useful use leads to positive impacts on performance mechanisms and favorable outcomes in performance.

Excessive use of these networks causes a drop in academic performance as well as educational performance. Spending more time in these networks reduces the study of students. Also, since most students spend long hours of the night on these networks, they cannot attend the classrooms with enough concentration. As a result, spending too much time using virtual networks has a negative effect on students and disrupts their academic achievement and education.

Individuals who show failure may in the future develop an expectation of low achievement on similar tasks, especially if they believe that failure is caused by stable and uncontrolled causes, such as lack of ability. Once individuals believe they may fail in an upcoming task, they are more likely to engage in self-handicapped behavior. The cycle of failure → self-handicapping → failure can result in a gradual withdrawal of effort in college (or any domain), leading to complete withdrawal from activities. When students are concerned that they may be doing worse than their peers (although they are not assessed as academically incompetent), they are more likely to self-handicapping (Schwinger et al., 2022).

## Conclusion

The purpose of the current study was to investigate the effect of social networks on academic self-handicapping with the mediating role of self-regulatory learning strategies and academic achievement among EFL students. This study showed that social networks have a significant impact on self-handicapping in language students of Tabriz University, second social networks have a substantial impact on self-handicapping with the mediating role of self-regulated learning strategies in language students of Tabriz University, and third-social networks have a significant impact on self-handicapping with a mediating role of academic progress in language students of Tabriz University. Further investigation and experimentation into social networks are strongly recommended. In addition, studies show that self-handicapping is successful in helping individuals divert the assessments of others from attributing weak abilities to failure. Because self-handicapping behavior undermines achievement and can lead to long-term withdrawal from activities (such as school and college), parents and teachers must discourage self-handicapping and avoid behavior that may encourage it.

In the present study, there were limitations such as not investigating the role of factors such as academic motivation, self-efficacy, and beliefs related to academic affairs. Generalizing the results of the larger society and the entire country’s students should be done with caution. According to the findings, it is suggested: National media should consider programs as informal education to inform about the harmful consequences of improper use of social networks. The Educational Research and Planning Organization of the country and the textbook authoring office and institutions related to the production of curriculum content should compile content to improve students’ awareness about using social networks correctly. School managers should organize educational workshops to inform the student community so that students know the disadvantages and advantages of virtual networks and can make good use of social networks to advance their education. Administrators, teachers, and parents of students should cooperate to control students’ optimal use of social networks. Teachers should inform students about the benefits and goals of using self-regulated learning strategies. Teachers should teach students how to use self-regulated learning strategies in the learning process in appropriate extracurricular opportunities.

## Data availability statement

The raw data supporting the conclusions of this article will be made available by the authors, without undue reservation.

## Author contributions

All authors listed have made a substantial, direct, and intellectual contribution to the work and approved it for publication.

## Conflict of interest

The authors declare that the research was conducted in the absence of any commercial or financial relationships that could be construed as a potential conflict of interest.

## Publisher’s note

All claims expressed in this article are solely those of the authors and do not necessarily represent those of their affiliated organizations, or those of the publisher, the editors and the reviewers. Any product that may be evaluated in this article, or claim that may be made by its manufacturer, is not guaranteed or endorsed by the publisher.
